# Antibacterial and Antitumoral Potentials of Phytosynthesized Silver/Silver Oxide Nanoparticles Using Tomato Flower Waste

**DOI:** 10.3390/ijms25189871

**Published:** 2024-09-12

**Authors:** Simona Marcu Spinu, Mihaela Dragoi Cudalbeanu, Ionela Avram, Radu Claudiu Fierascu, Petronela Mihaela Rosu, Ana-Maria Morosanu, Carmen Laura Cimpeanu, Narcisa Babeanu, Alina Ortan

**Affiliations:** 1 Faculty of Land Reclamation and Environmental Engineering, University of Agronomic Sciences and Veterinary Medicine of Bucharest, 59 Marasti Blvd., 011464 Bucharest, Romania; simona.spinu@fifim.ro (S.M.S.); alina.ortan@fifim.ro (A.O.); 2 Department of Genetics, University of Bucharest, 1-3 Aleea Portocalelor, 060101 Bucharest, Romania; ionela.avram@unibuc.ro; 3 National Institute for Research & Development in Chemistry and Petrochemistry–ICECHIM Bucharest, 202 Splaiul Independenței, 060021 Bucharest, Romania; fierascu.radu@icechim.ro; 4 Faculty of Chemical Engineering and Biotechnology, National University of Science and Technology Politehnica Bucharest, 1-7 Gheorghe Polizu St., 011061 Bucharest, Romania; 5 Faculty of Veterinary Medicine, University of Agronomic Sciences and Veterinary Medicine of Bucharest, 59 Marasti Blvd., 011464 Bucharest, Romania; petronela.rosu@fmvb.usamv.ro; 6 Institute of Biology Bucharest, Romanian Academy, 060031 Bucharest, Romania; anamaria.morosanu@ibiol.ro; 7 Faculty of Biotechnologies, University of Agronomic Sciences and Veterinary Medicine of Bucharest, 59 Marasti Blvd., 011464 Bucharest, Romania; narcisa.babeanu@usamv.ro

**Keywords:** tomato waste, phytochemical profile, metallic nanoparticles, pathogenic bacteria, HeLa tumor cells, HT29 tumor cells

## Abstract

This study presents the phytosynthesis of silver-based nanoparticles using tomato flower waste extracts for the first time in the literature. The determination of total polyphenolic and flavonoid contents in the extracts showed high gallic acid equivalents (6436–8802 mg GAE/kg dm) and high quercetin equivalents (378–633 mg QE/kg dm), respectively, dependent on the extraction method. By the Ultra Performance Liquid Chromatography technique, 14 polyphenolic compounds were identified and quantified in the tomato flower waste extracts. The abundant phenolic compounds were caffeic acid (36,902–32,217 mg/kg) and chlorogenic acid (1640–1728 mg/kg), and the abundant flavonoid compounds were catechin (292–251 mg/kg) and luteolin (246–108 mg/kg). Transmission electron microscopy of the nanoparticles revealed a particle size range of 14–40 nm. Fourier Transform infrared spectroscopy and X-ray diffraction studies confirmed the phytosynthesis of the silver/silver oxide nanoparticles. These findings hold significant results for the antibacterial and antitumoral potential applications of the obtained nanoparticles, opening new areas for research and development and inspiring further exploration. The impact of this research on the field of metallic nanoparticle phytosynthesis is substantial, as it introduces a novel approach and could lead to significant advancements in the field.

## 1. Introduction

Tomatoes, globally distributed plants, have origins in America (the Aztec city of Tenochtitla—now Mexico City) and have been imported to Europe by Spanish colonists since the 16th century [1]. Today, tomatoes are considered fruits according to botanical classifications, and vegetables according to culinary classifications, being cultivated both in greenhouses and open agricultural lands. Although tomatoes can be found on the market in the form of fresh tomatoes, they are also available in a wide variety of by-products (dried tomatoes, canned tomatoes, tomato pastes, ketchup, tomato purees, tomato juice, etc.).

Globally, between 2018 and 2022, an average of 4,972,265.6 ha of tomato areas were cultivated per year, with an average of 184,940,640.7 tons of tomatoes produced per year [2]. In Europe, 415,163.4 ha of tomato areas were cultivated on average per year, and 22,833,989.89 tons of tomatoes were produced on average per year [2]. The production of tomatoes, at the European level, increased in 2021, followed by a significant decrease in 2022. The European countries with the highest production of tomatoes are Turkey, Italy, and Spain, which are part of the top 10 producing countries at the global level. In Romania, between 2018 and 2022, an average of 438,686 tons of tomatoes were produced per year, and 19,904 ha of land was cultivated [2].

Considering the high production of tomatoes, a significant amount of vegetable waste results from both tomato crops and post-harvest industrial processing. The aerial biomass formed by leaves, suckers, bunches, stems, and dried flowers after the ripening of the fruit constitutes vegetable agricultural waste. The waste from tomato crops that results at the end of the cultivation cycle has become the subject of numerous studies aimed at the valorization of this important resource. For example, Añibarro-Ortega et al. (2020) recently studied the antioxidant and antimicrobial properties of tomato crop residues resulting from cuttings and biomass left after harvesting to point out the direction of their reuse in the agri-food sector [3]. Many studies have proven that through anaerobic digestion of tomato leaves and stems, pollution can be effectively reduced and bioenergy, i.e., biogas, can be produced [4,5,6]. Moreover, vegetable tomato plant waste is a potential sugar source for biorefineries, an aspect demonstrated by alkali-catalyzed extrusion methods followed by enzymatic hydrolysis [7], because of the high content of carbohydrates, lignin (acid-insoluble and acid-soluble), and ash. Carbohydrates, saponins, tannins, glycosides, phenols, coumarins, alkaloids, flavonoids, resins, and terpenoids are the main biologically active classes of compounds present in both tomato leaves and stems [8]. For maximum utilization of tomato plant waste, they were used as a growing medium [9] and as a substrate (compost) [10,11], indicating favorable results.

A tomato plant has small yellow flowers in the form of clusters, which contain pistils and stamens (male and female reproductive organs). The tomato flower is the key element in the formation of the fruit, and for the maximum production of tomatoes, as few aborted or unfertilized flowers as possible are needed to minimize negative economic effects. In the case of a tomato plant, whose flowers are hermaphrodite, pollination is carried out predominantly with its own pollen and can take place on the same flower as soon as the pollen has been formed. In greenhouses, the pollination of tomato flowers can be achieved by various methods such as insect pollination, artificial pollination, hormonal pollination, and advanced techniques using drones or robots [12].

Metal nanoparticles continue to interest researchers because of their diverse properties [13,14,15]. There are various studies that attest to the beneficial effect of metal NPs (copper [16,17], zinc [18,19,20], silver [21,22,23,24,25,26,27], selenium [28,29], and iron [30]) in improving tomato plant growth and yield, as well as increasing life span. At the same time, there are various studies that prove the bio-medical applicability of metal NPs phytosynthesized using different parts of tomato plants such as leaves [31,32], fruits [33,34,35,36,37,38,39], seeds [36], and entire plants [40]. In these studies, biological activities such as antibacterial, antifungal, antitumoral, antioxidant, and antidiabetic are highlighted.

Silver and silver oxide nanoparticles are known for their unique properties, being used in numerous fields including electrochemistry and electronics (they improve the efficiency and functionality of devices), catalysis (their ability to oxidize makes them valuable for various chemical reactions and industrial processes), energy storage systems and renewable energy (sensors, photovoltaic cells), and optics (integral switching devices and optical data storage systems). In the medical and pharmaceutical field, they can be functionalized to attach to specific biological molecules, chemotherapy, and antibiotic treatment, and in cosmetics, their beneficial properties are used in skin care products [41,42,43,44,45,46].

The present study proposes a new approach for the valorization of tomato waste produced at the end of the crop cycle, in particular, the dried tomato flowers that remain after the ripening of the fruit, for the phytosynthesis of silver oxide nanoparticles with therapeutic potential. To date, no studies have focused on the valorization of dried tomato flowers, harvested after the fruit has reached maturity, to our knowledge. In addition, no studies have demonstrated the biological activities of silver-based nanoparticles phytosynthesized using tomato flower waste extracts, specifically from dried tomato flowers left at the end of the development and ripening cycle of the fruits. Therefore, the aim of this study was to demonstrate the therapeutic potential (antibacterial and antitumoral) of phytosynthesized silver-based nanoparticles using tomato flower waste extract.

## 2. Results

### 2.1. Effect of Extraction Method on Extract Yield

**Figure 1:**
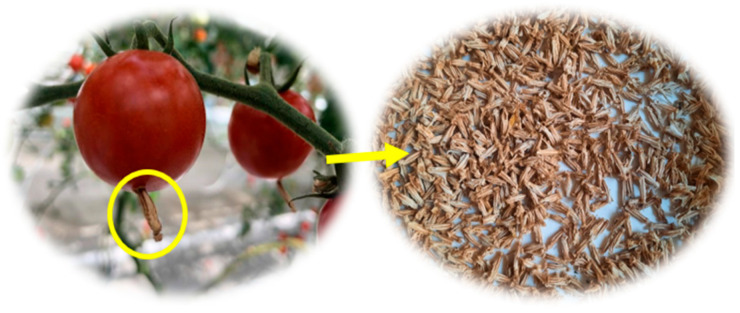
Tomato flower waste collected from the University of Agronomic Sciences and Veterinary Medicine of Bucharest (USAMV) Research Greenhouse.

The tomato plant waste, i.e., dried flowers left after the ripening cycle of *Lycopersicon esculentum* (Cheramy RZ F1 hybrid) fruit, was oven-dried and then subjected to extraction (Figure 1).

Following the extraction of the plant material through the three selected methods, ultrasound-assisted extraction (UAE), microwave-assisted extraction (MAE), and cascade extraction (CASE), the extraction yield of tomato flower waste (TFW) was determined (Table 1). All the investigated extraction methods showed high values of TFW, varying between approx. 20% for TFWCAS and approx. 27% for TFWMW.

### 2.2. Effect of the Extraction Method on the Total Polyphenolic Content (TPC) and Total Flavonoid Content (TFC)

**Figure 2:**
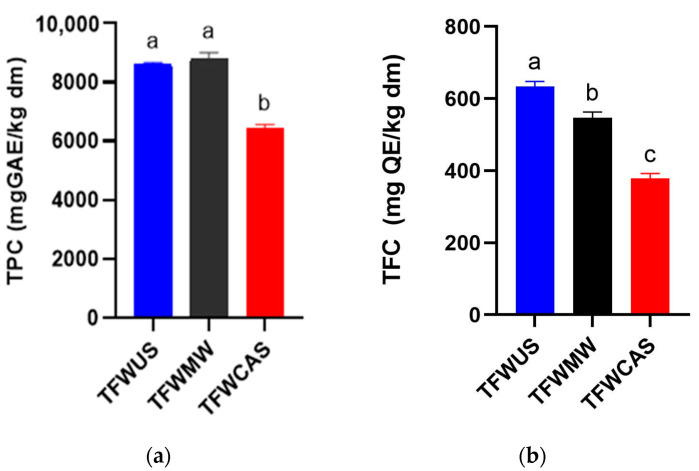
Total phenolic content (**a**) and total flavonoid content (**b**) of tomato flower waste extracts obtained by MAE, UAE, and CASE. The letters a–c indicate statistically significant differences detected by ANOVA (*p* < 0.05).

Tomato flower waste extracts revealed a significant total polyphenolic content (TPC, Figure 2a) ranging from approx. 6436 mg GAE/kg dm (TFWCAS) up to 8802 mg GAE/kg dm (TFWMW). The highest value was obtained for TFWMW, which is not statistically significant compared to TFWUS, but it was statistically different compared with TFWCAS.

Regarding total flavonoid content (TFC), the TFW extracts revealed values around 378 mg QE/kg dm (TFWCAS) and 633 mg QE/kg dm (TFWUS), as shown in Figure 2b. There were statistically significant differences in the TFC obtained from TFW using all the extraction methods, confirming the influence of the extraction method on both TPC and TFC.

### 2.3. Quantitative Analysis of Polyphenolic Compounds Present in Tomato Flower Waste Extracts by UPLC

**Figure 3:**
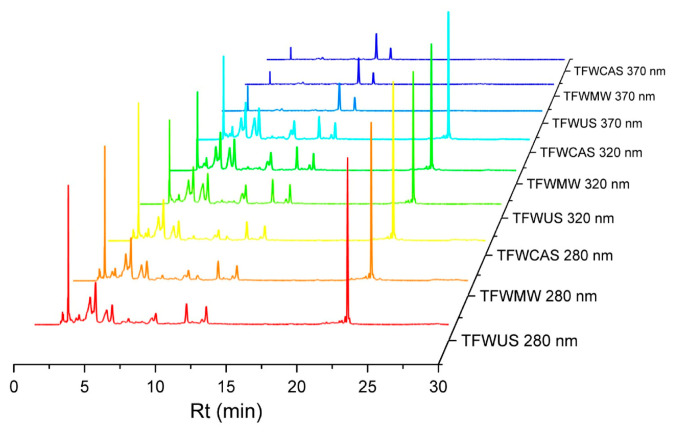
Chromatogram of tomato flower waste extracts obtained by different extraction methods. US, ultrasonic-assisted extraction; MW, microwave-assisted extraction; and CAS, cascade extraction.

The polyphenolic compounds in all TFW extracts, TFWUS, TFWMW, and TFWCAS, were identified by the Ultra Performance Liquid Chromatography (UPLC) system. Figure 3 shows the UPLC chromatogram of TFW extracts detected at 280 nm, 320 nm, and 370 nm. The polyphenolic compounds detected were initially compared with the Photodiode array (PDA) spectra of targeted reference compounds. The targeted reference polyphenolic compounds were gallic acid, caffeic acid, chlorogenic acid, catechin, epicatechin, p-coumaric acid, ferulic acid, isoquercetin, rosmarinic acid, naringin, myricetin, luteolin, quercetin, and naringenin. A total of 14 polyphenolic compounds were identified in the TFW extracts (Table 2).

The total content of the identified phenolic acids was approx. 39,571 mg/kg, 40,015 mg/kg, and 34,898 mg/kg, in the case of the TFWUS, TFWMW, and TFWCAS extracts, respectively. The compound with the highest content was caffeic acid (over 38,720 mg/kg—TFWMW, 36,902 mg/kg—TFWUS, and 32,217 mg/kg—TFWCAS), followed by chlorogenic acid (1728 mg/kg—TFWCAS and 1640 mg/kg—TFWUS). In comparison with the UAE and CASE extractions, minimal amounts of chlorogenic acid were found in the MAE extraction. The TFWMW extract contained only 106 mg/kg of chlorogenic acid.

The total content of the identified flavonoids was approx. 866 mg/kg, 711 mg/kg, and 643 mg/kg, in the case of TFWUS, TFWMW, and TFWCAS, respectively. The highest content was found to be catechin (approximately 292 mg/kg for TFWUS, 288 mg/kg for TFWMW, and 253 mg/kg for TFWCAS), followed by luteolin, registering approximately 246 mg/kg in the TFWUS extract, 108 mg/kg for TFWCAS, and 105 mg/kg for TFWMW. Similar results were observed for the flavonoids isoquercetin (61.61 mg/kg—TFWUS, 18.97 mg/kg—TFWMW, and 15.50 mg/kg—TFWCAS) and naringin (45.37 mg/kg—TFWUS, 28.05 mg/kg—TFWMW, and 26.51 mg/kg—TFWCAS). Similar to chlorogenic acid, the flavonoid epicatechin minimum content was found in the MAE extraction (TFWMW—57.95 mg/kg, TFWUS—78.11 mg/kg, and TFWCAS—77.10 mg/kg).

Additionally, the TFWMW extract contained the following compounds: gallic acid, p-coumaric acid, ferulic acid, rosmarinic acid, myricetin, quercetin, and naringenin, in slightly higher concentrations than the TFWUS and TFWCAS extracts. The CASE extraction revealed the lowest content of the identified polyphenolic compounds using the UPLC technique.

### 2.4. Evaluation of the Antioxidant Activity of TFW Extracts 

In the present study, the antioxidant activity of TFW extracts was evaluated by three different methods (DPPH, ABTS, and FRAP). The studied TFW extracts demonstrated a high capacity to scavenge the considered free radicals (Table 3).

The results of the DPPH assay test, ranging from approx. 519 to 655 mg Ascorbic Acid Equivalent (AAE) per kg of dry matter (dm), showed differences in the antioxidant capacity of the investigated samples, depending on the extraction method used. The DPPH assay results demonstrated significant antioxidant activity of the dried TFW samples.

For ABTS scavenging activity, the results ranged from 15,379 to 16,783 AAE/kg dm. TFWCAS led to statistically significantly lower results than TFWMW. Regarding the FRAP assay, the antioxidant potential ranged from 4594 mg AAE/kg dm for TFWCAS to 5880 mg AAE/kg dm for TFWMW.

**Figure 4:**
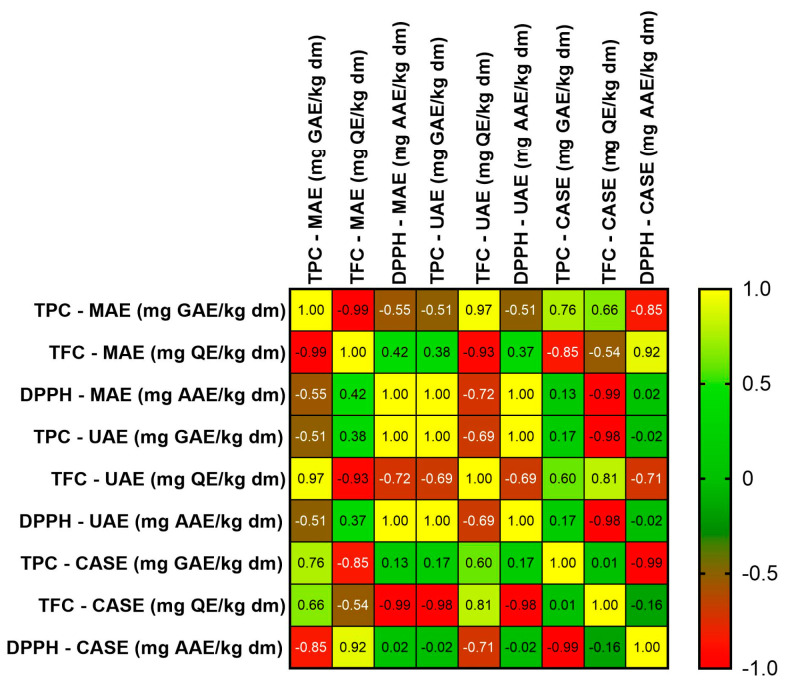
Correlation coefficient matrix among TPC, TFC, and the DPPH assay.

After analyzing the correlations among TPC, TFC, and the antioxidant potential tested by the DPPH method, using the Pearson correlation coefficient, direct and indirect relationships among these parameters were observed, as illustrated in the Figure 4.

Strong positive correlations were identified between DPPH assay values and TPC (Pearson correlation coefficient = 1.00, *p* < 0.01) in the case of the TFWUS extract. A strong negative correlation was found between TFC and TPC (Pearson correlation coefficient = −0.99) in the case of the TFWMW extract and between DPPH assay values and TPC (Pearson correlation coefficient = −0.99) for the TFWCAS extract.

### 2.5. Silver-Based Nanoparticles

The phytosynthesis of silver-based nanoparticles was performed using TFW extracts, which served as both reducing and capping agents.

The yield of NPs is presented in Table 4. The TFWMW-NPs sample exhibited the highest yield of NPs, i.e., a yield of 34.8%, followed by the TFWCAS-NPs sample, which exhibited a yield of 32.2%. The TFWUS-NPs sample obtained a lower yield of NPs (29.3%).

### 2.6. Characterization of Silver-Based Nanoparticles

#### 2.6.1. Preliminary Evaluation by UV-Vis Spectrometry

**Figure 5:**
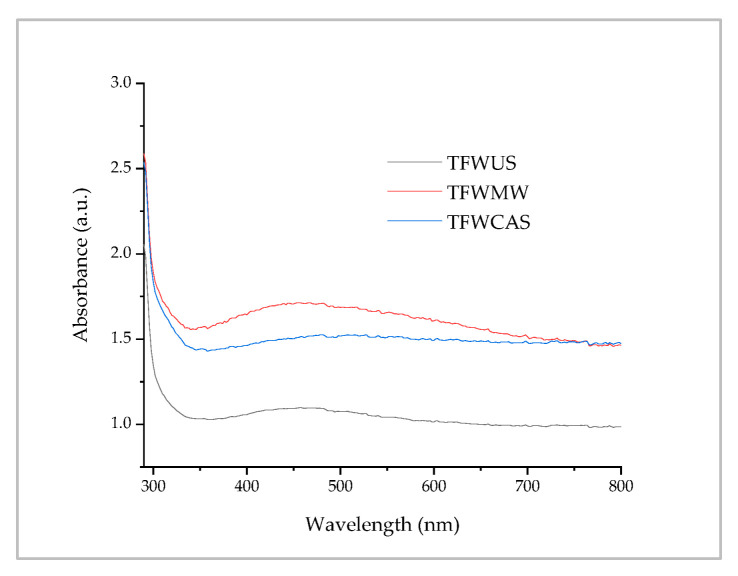
Absorption spectra of the phytosynthesized NPs.

The phytosynthesis of nanoparticles was preliminarily evaluated by UV-Vis spectrometry. This helped to identify the formation of metal nanoparticles because the phenomenon of surface plasmon resonance leads to the appearance of specific peaks of nanoparticles in various states including metal oxides, zero-valent metals, etc. The obtained spectra are presented in Figure 5.

The UV-Vis absorption spectra revealed a relatively low-intensity shoulder in the region of interest (400–500 nm) where AgNPs present specific peaks. The low intensity and the insufficient definition of the peak could be attributed to the presence of silver oxide in the samples (a species that does not exhibit specific maxima in this region).

#### 2.6.2. FTIR Spectroscopy

**Figure 6:**
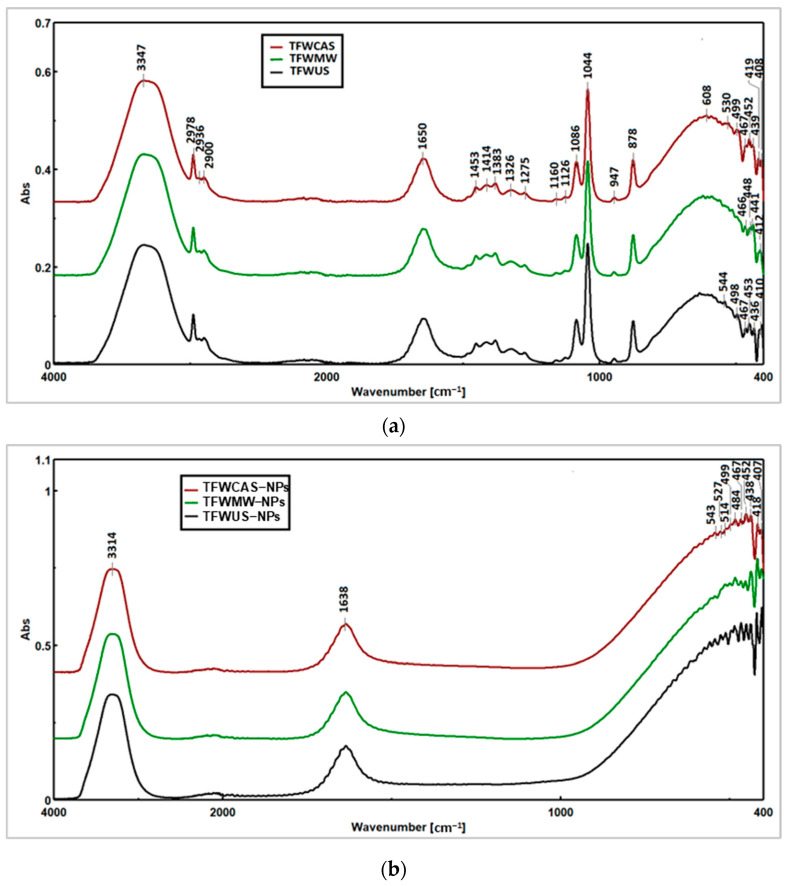
FTIR spectra of (**a**) TFW extracts and (**b**) phytosynthesized NPs.

FTIR spectroscopy analysis was used to compare the TFW extracts (before the reaction, without AgNO_3_) with the NPs formed (after the reaction with AgNO_3_), and the obtained spectra are presented in Figure 6.

Both FTIR spectra (TFW extracts and NPs) showed two significant stretching bands at 3316–3347 cm^−1^(due to the N–H stretching vibration of amines or the O−H stretching vibration of polymeric hydroxyl groups, which are located in close proximity) and at 1635–1650 cm^−1^ (due to the C=C and C−C stretching vibrations of aromatic rings). The stretching vibrations of Ag−Ag, Ag−O−Ag, Ag−O, and O−Ag−O were observed at peaks between 400 and 600 cm^−1^ (Figure 6b).

Figure 6a shows the absorption spectra of TFW extracts obtained in the range of 4000–400 cm^−1^. In the region of 2978–2890 cm^−1^, the –CH, –CH_2_, and –CH_3_ stretching vibrations are derived from carbohydrates and sugars present in the TFW extracts. In addition, the phenolic C−O stretching vibration was observed at 1276 cm^−1^. The C−N bond of the amine or the C−O bond of the primary alcohol is caused by stretching vibrations at 1044 cm^−1^. The peaks between 636 and 1454 cm^−1^ are attributed to C=C–C aromatic ring stretching (1454 cm^−1^) and several aromatic out-of-plane bending C–H (636–948 cm^−1^) and in-plane bending (948–1276 cm^−1^).

#### 2.6.3. XRD

**Figure 7:**
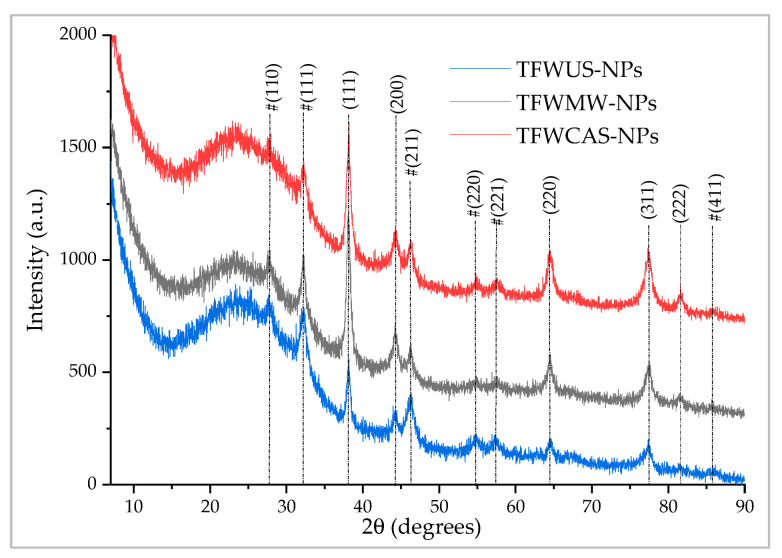
XRD for NPs phytosynthesized by tomato flower waste extracts.

The XRD technique was used in order to identify the phase composition of the obtained materials. The identification of the diffraction peaks was based on the comparison with ICDD entries 01-071-4613 (Ag) and 01-078-5867 (Ag_2_O, marked with # in Figure 7).

Crystallite size was determined using the Debye–Scherrer equation:

Dp = (K × λ)/(β × cosθ)(1)

where Dp represents the average size of the crystallites, K represents the Scherrer constant (for cubic structures, K = 0.94), β represents the width at half-height of the diffraction maximum, θ represents the Bragg angle, and λ represents the wavelength—1.54059 Å, in our case.

The results for TFW-NPs are presented in Table 5:

The XRD analysis showed the co-existence of the following two phases: silver nanoparticles (AgNPs) and silver oxide nanoparticles (Ag_2_O-NPs). Their crystallite sizes, determined using the Scherrer equation, revealed a similar trend for both nanoparticle species, with the TFWUS-NPs leading to the largest crystallites and TFWCAS-NPs to the smallest.

#### 2.6.4. Morphology and Size Distribution of Silver/Silver Oxide Nanoparticles

**Figure 8:**
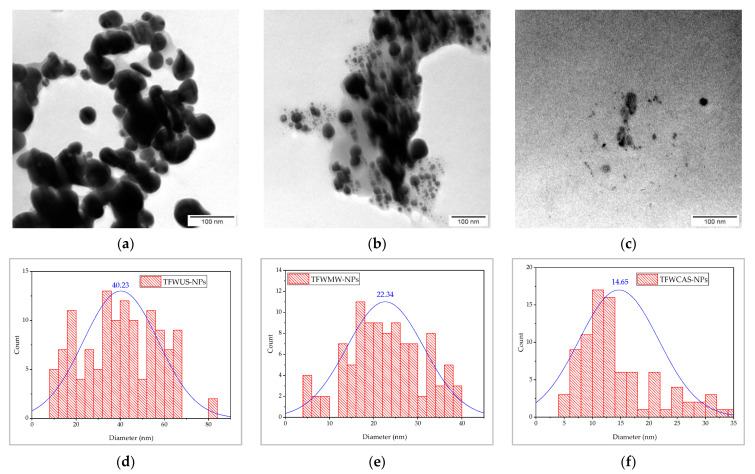
TEM images (**a**–**c**) and particle size distribution histograms (**d**–**f**) of the TFWUS-NP, TFWMW-NP, and TFWCAS-NP samples.

The morphology and size of the Ag/Ag_2_O-NPs formed by TFW extracts were determined by Transmission electron microscopy (TEM) analysis, as shown in Figure 8. The average particle diameters for the TFWUS-NP, TFWMW-NP, and TFWCAS-NP samples were found to be around 40 nm, 22 nm, and 14 nm, respectively. The TEM images (Figure 8a–c) indicate the presence of mostly spherical nanoparticles, with the presence of other morphologies.

### 2.7. Antibacterial Potential of Tomato Flower Extracts and Silver/Silver Oxide Nanoparticles

**Figure 9:**
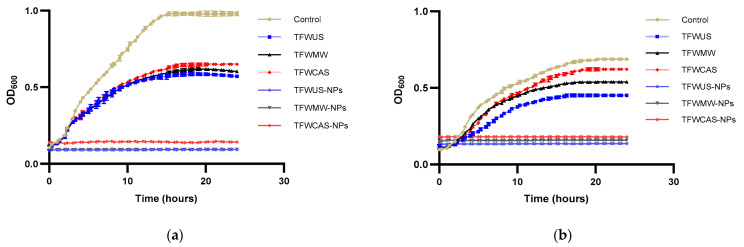
Time-kill kinetics curves of (**a**) *E. coli* and (**b**) *S. aureus* treated with the TFW extracts and NPs. Data are presented as means ± standard deviations (n = 3).

The antimicrobial activities of the TFW extracts and NPs against pathogenic bacteria, including *E. coli* (Gram-negative) and *S. aureus* (Gram-positive), were carried out through the time-kill kinetics assay. The results showed that both the TFW extracts and NPs exhibited greater antimicrobial potential against *E. coli* than against *S. aureus* (Figure 9). The NPs exhibited higher antimicrobial potential than the TFW extracts. The utilization of TFW extracts in the phytosynthesis of Ag/Ag_2_O-NPs was a success. The antimicrobial potential of the Ag/Ag_2_O-NPs was significantly higher than if were used only TFW extracts against pathogenic bacteria. The TFW extracts show greater potential against Gram-negative bacteria (*E. coli*) than against Gram-positive bacteria (*S. aureus*). In both cases of antimicrobial potential evaluation of the TFW extracts, TFWUS was more active than the TFWMW and TFWCAS extracts. Regarding the antimicrobial potential of NPs against *E. coli*, TFWUS-NPs and TFWMW-NPs had the highest antimicrobial potential (equal), followed by TFWCAS-NPs. On the other hand, the antimicrobial potential of NPs against *S. aureus* showed the following results: TFWUS-NPs presented the highest antimicrobial potential, followed by TFWMW-NPs and TFWCAS-NPs.

### 2.8. Antitumoral Potential of Tomato Flower Extracts and Silver/Silver Oxide Nanoparticles

**Figure 10:**
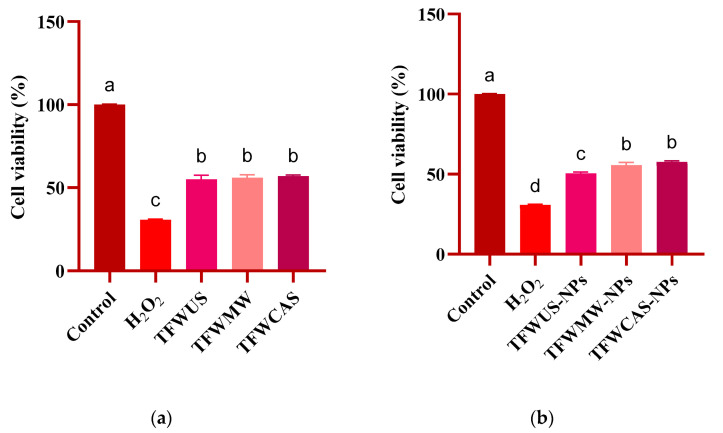
MTT assessment of HeLa cells treated with the (**a**) TFW extracts and (**b**) NPs after 24 h. Data are presented as means ± standard deviations (n = 3). a–d indicate a significant difference, *p* ≤ 0.0001 compared with the control, calculated using one-way ANOVA.

**Figure 11:**
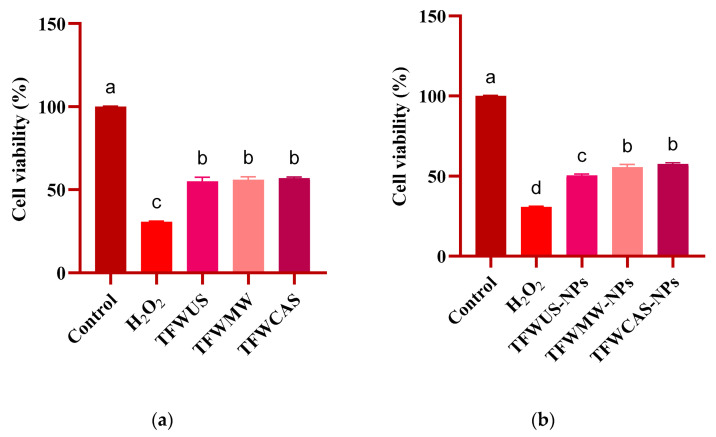
MTT assessment of HT29 cells treated with the (**a**) TFW extracts and (**b**) NPs after 24 h. Data are presented as means ± standard deviations (n = 3). a–c indicate a significant difference, *p* ≤ 0.0001, compared with the control, calculated using one-way ANOVA.

Our review of the literature review identified no studies regarding the evaluation of the antitumoral potential of NPs phytosynthesized using TFW extracts in cervical tumor (HeLa) and colon tumor (HT29) cell lines, to our knowledge. The antitumoral potential evaluation of the TFW extracts and NPs showed a significantly reduced viable number of HeLa (Figure 10) and HT29 (Figure 11) cells. For both tumor cell lines studied, the TFW extracts showed similar results, with a cell viability of around 55% (Figure 10a and Figure 11a). Regarding the NP (TFWUS-NPs, TFWMW-NPs, and TFWCAS-NPs) results against the studied tumor cell lines, the NPs showed higher antitumoral potential against HeLa cells (Figure 10b) than against HT29 cells (Figure 11b). The mentioned results could be ascribed to the greater sensitivity of HeLa cells to the silver-based NPs compared with HT29 cells. In both cases, TFWUS-NPs had the highest reduced viable number of tumor cells (50.49%—HeLa, 62.45%—HT29).

**Figure 12:**
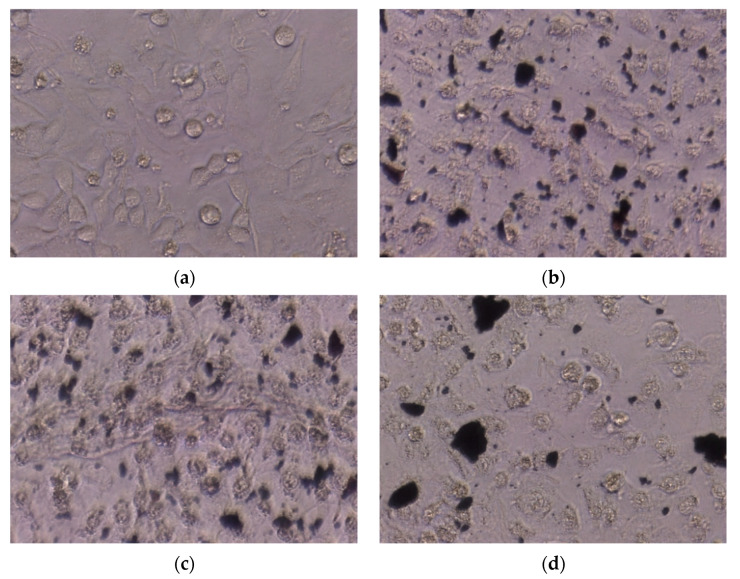
Cell morphology of (**a**) untreated HeLa cells (control) and HeLa cells treated with (**b**) TFWUS-NPs, (**b**,**c**) TFWMW-NPs, and (**d**) TFWCAS-NPs.

**Figure 13:**
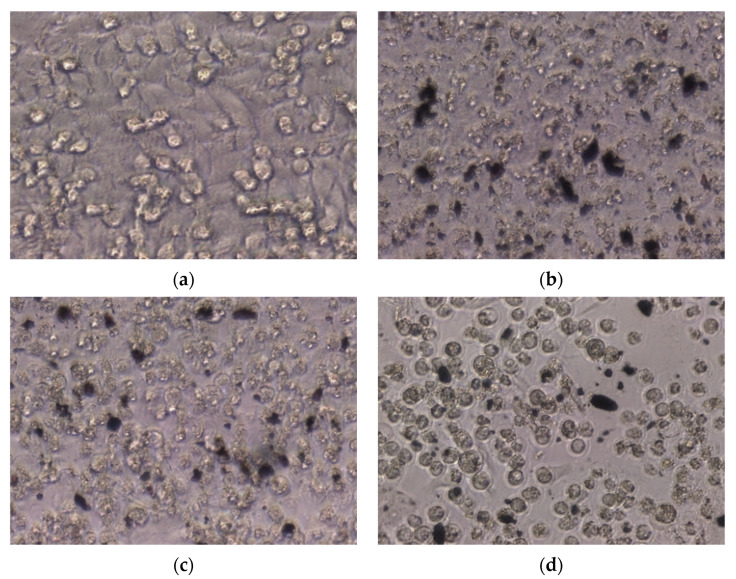
Cell morphology of (**a**) untreated HeLa cells (Control) and HT29 cells treated with (**b**) TFWUS-NPs, (**b**,**c**) TFWMW-NPs, and (**d**) TFWCAS-NPs.

Upon microscopic analysis, the HeLa (Figure 12) and HT29 (Figure 13) cells treated with NPs exhibited dead tumor cells, showing cell deformation such as contraction, rounding, and detachment from adjacent cells. In contrast, the untreated (control) cells displayed a normal, round characteristic nucleus.

## 3. Discussion

The extraction methods for bioactive compounds are diverse, encompassing conventional as well as advanced methods, such as those employed in this study. Each method is adapted depending on the nature of the compounds of interest and also on the plant material used. Selecting the right extraction method is essential to obtain a high extraction yield and also to ensure the quantity and quality of the compounds of interest. The extraction yield is primarily influenced by temperature, extraction time, and the solvent used for extraction. Comparing the extraction yields for the three methods investigated, MAE led to the highest extraction yield of TFW, followed by UAE and CASE, probably because of its ability to heat the solvent and plant matrix rapidly, therefore enhancing mass transfer, breaking down cell walls, and releasing the bioactive compounds more efficiently. We observed that CASE led to a low yield compared with the other two selected methods, likely because of the sequential application of the two extraction methods, which probably led to partial degradation of sensitive compounds during the extended processing time. As presented in the Materials and Methods Section, in this study, a hydroalcoholic solvent with a 50% (*v*/*v*) aqueous ethanol concentration was used to maximize the extraction yield across the selected methods. The obtained results are in accordance with the results reported by other researchers. For example, in the case of waste from sugar beet leaves, the extraction yield in the case of MAE was higher than the case of UAE using 50% ethanol as an extraction solvent [47]. At the same time, the extraction yield of TFW is similar, but even higher than the extraction yield obtained by classical ethanolic extraction from the aerial parts of tomatoes such as leaves, stems, and suckers [3].

The evaluation of total polyphenol content and flavonoid content represents the first investigation step for vegetable waste extracts, in an attempt to prove their promising biological activities, such as anti-inflammatory, antioxidant, anti-diabetic, antitumoral, antibacterial, and anti-aging effects [48,49,50,51].

Numerous studies have proven the efficiency of MAE for the quantitative extraction of polyphenols, compared with other extraction techniques, in the case of different plant materials. For example, ethanol extracts from jackfruit pulp obtained by microwave-assisted extraction showed significantly better results regarding TPC, DPPH, and ABTS radical scavenging activities compared with those obtained by ultrasound-assisted extraction and ultrasound-microwave-assisted extraction [52]. Afoakwah et al. [53] reported similar results for the same extraction technique, which proved effective in the extraction of polyphenols from the ethanolic extracts of Jerusalem artichoke tubers and also identified a high antioxidant activity compared with the values obtained from liquid–liquid extraction or ultrasound-assisted extraction.

Usually, a high flavonoid content in plant wastes is obtained using ultrasound-assisted extraction [54,55]. For example, Liao et al. [56] reported higher flavonoid content using ultrasound-assisted extraction from peanut shell wastes. These already reported results are in accordance with the higher flavonoid content obtained by ultrasound-assisted extraction compared with microwave-assisted extraction in the present investigation.

The phytochemical screening of TFW extracts, as well as the evaluation of their antioxidant potential, indicated that MAE and UAE proved to be suitable extraction techniques, leading to the highest contents of polyphenols and flavonoids and the best antioxidant activity. The highest content of total phenolic acids was TFWMW (40,015 mg/kg) > TFWUS (39,571 mg/kg) > TFWCAS (approx. 34,898 mg/kg). On the other hand, the highest content of total flavonoids was TFWUS (approx. 866 mg/kg) > TFWMW (711 mg/kg) > TFWCAS (approx. 643 mg/kg).

The TFW hydroalcoholic extracts revealed the capacity to scavenge DPPH radicals, proving their antioxidant activity. TFWMW revealed the highest value of approx. 656 mg AAE/kg dm, indicating that the MAE method is significantly more efficient compared with the UAE and CASE methods. The statistical analysis demonstrated that there were no significant differences between TFWUS (approx. 523 mg AAE/kg dm) and TFWCAS (approx. 519 mg AAE/kg dm) (*p* > 0.05). The TFWMW extract showed higher antioxidant activity than the TFWUS and TFWCAS extracts, which can be attributed to the higher content of phenolic acids.

Regarding the ABTS assay, the scavenging activity followed the order TFWMW > TFWUS > TFWCAS. There were no statistically significant differences between TFWMW and TFWUS (*p* > 0.05). In the case of the FRAP assay, TFWMW also revealed the highest antioxidant potential; the antioxidant potential varied in the following order TFWMW > TFWUS > TFWCAS. Similar results, comparing the extraction method, regarding DPPH scavenging activity and the FRAP assay were obtained in the case of aerial parts of sea fennel plants, where MAE revealed higher inhibition values than UAE. Moreover, the highest value for free-radical scavenging activity using the FRAP assay was achieved in a study conducted by Veršić Bratinčević et al. when they used MAE extraction at 500 W [57]. Using aqueous ethanol extracts (50%) in the case of *Lavandula* extracts from stems, leaves, and fruit, a higher antioxidant potential was revealed by MAE, compared with UAE, when tested with the ABTS and DPPH assays [58]. These results confirmed the optimal use of the solvent and of the extraction method. Regarding the antioxidant activity assessment by the three different methods investigated (DPPH, ABTS, FRAP), significant differences (*p* < 0.05) were observed in the FRAP assay among all studied extracts.

The present study aimed at the phytosynthesis, characterization, and biological potential evaluation of NPs. The NPs were phytosynthesized by TFW extracts, and the structural characteristics were investigated using a range of analytical techniques such as UV-Vis, FTIR, XRD, and TEM. The preliminary phytochemical screening described above for the TFW extracts showed a high content of polyphenols and flavonoids. Also, the UPLC analysis indicated the presence of the following polyphenolic compounds: gallic acid, caffeic acid, chlorogenic acid, catechin, epicatechin, p-coumaric acid, ferulic acid, isoquercetin, rosmarinic acid, naringin, myricetin, luteolin, quercetin, and naringenin. These compounds, especially flavonoids, present in the TFW extracts can be responsible for the NPs’ phytosynthesis and stabilization [59].

The accumulation of phenolic compounds is significantly influenced by tomato cultivars. Environmental factors such as temperature, solar radiation, weather variation and anomalies, light exposure, and agricultural practices such as irrigation methods and nitrogen supply are factors with a role in determining the level of phenolic compound accumulation. The main compound identified in the TFW extracts was caffeic acid followed by chlorogenic acid. These compounds were also identified in several studies targeting tomato plant valorization. Chlorogenic acid was also abundant in tomato leaf extracts of several cultivars [60].

The analysis of the UV-Vis spectra supports the hypothesis of the formation of silver-based NPs, by the presence of the peak associated with the surface plasmonic resonance in the 400–500 nm range. Often, UV-Vis spectrometry can be applied to evaluate the sizes of NPs by determining the position of the specific peak. However, these values should not be considered definitive dimensions, since the position of the specific peaks of phytosynthesized silver-based NPs may undergo a bathochromic shift from its “true” position because of the influence of a series of factors, such as aggregation of NPs into larger clusters and the presence of different phytoconstituents. As such, NP sizes must be further confirmed using other techniques such as XRD or TEM.

FTIR spectroscopy was used to identify the functional groups responsible for the phytosynthesis of NPs. When infrared radiation interacts with TFW extracts or NP samples, the bonds within molecules vibrate at characteristic frequencies, leading to absorption peaks on the FTIR spectrum, each corresponding to specific functional groups. The discovered stretching vibrations of Ag–Ag, Ag–O–Ag, Ag−O, and O−Ag−O were also identified in other studies previously reported in the literature [61,62,63,64]. Moreover, the absorption spectra of the TFW extracts were similar and showed characteristic peaks. The identified C–O stretching vibration was attributed to pyran, typical of flavonoid C-rings [65]. The analysis indicated that the functional groups present in the FTIR spectra of the TFW extracts and absent in the FTIR spectra of the NPs were involved in the reduction of Ag^+^ to Ag^0^ and stabilization of NPs.

In concordance with TEM analysis, almost all the TFW-NPs were spherical in shape. Few NPs were observed in agglomerated structures, which is a characteristic of phytosynthesized NPs using plant extracts [66]. The TEM morphology and size results are in good concordance with the XRD results.

Numerous studies in the literature describe the antimicrobial and antitumoral potentials of Ag-based NPs phytosynthesized by plant extracts [67,68,69,70,71,72,73]. Ag-based NPs disrupt cell membranes and damage intracellular structures [74,75].

In this study, the antimicrobial potential of silver-based NPs was evaluated against *E. coli* and *S. aureus*, and the results presented insignificant differences. NPs inhibited approximately 100% of bacteria cells. NPs showed good antimicrobial potential because of their positive charge and small size [76].

Differences were observed in the antibacterial potential of the TFW extracts. The TFW extracts inhibited approximately 50% of *E. coli* bacteria cells and between 60 and 85% of *S. aureus* bacteria cells. Thus, the TFWUS extract was the most active against *E. coli* and *S. aureus*. This could be attributed to the differences in chemical compositions and the action mechanism. The TFWUS extract contains the highest amount of flavonoids, which confirms the highest antimicrobial potential of both the extract and TFWUS-NPs. Flavonoids may have antimicrobial potential because of their ability to bind with intracellular and soluble proteins and bacterial cell walls [77].

The present study also investigated the antitumoral potentials of the TFW extracts and NPs against HeLa and HT29 tumor cell lines using the MTT assay. The antitumoral potential of the TFW extracts against HT29 cells was similar to the potential against HeLa cells. Mani et al. [78] reported that the HeLa and HT29 tumor cell lines treated with *M. koenigii* and *P. angustifolium* extracts showed similar potential against both tumor cell lines. The antitumoral potential may be attributed to polyphenolic antioxidant compounds, such as caffeic acid, ferulic acid, chlorogenic acid, p-coumaric acid, gallic acid, catechin, isoquercetin, luteolin, and quercetin, which have a significant role in inhibiting tumor cells [79].

The MTT results showed that the NPs were more active against HeLa cells than HT29 cells. Antitumoral potential also depends on NP concentration and the possible interaction with tumor cells. The antitumoral potential assay results evidenced that increasing the concentration of NPs significantly increased the reduced viable number of HeLa and HT29 tumor cells [80]. Also, the microscopic images showed that the NPs tend to agglomerate in the culture medium [81].

## 4. Materials and Methods

### 4.1. Tomato Flower Waste Material and Extract Preparation

The plant material used consisted of tomato plant waste and dried flowers left after the ripening cycle of *Lycopersicon esculentum* (Cheramy RZ F1 hybrid) fruit. These wastes from flowers can contain stigmas, styles, and parts of the anther cone. The tomato flower waste was collected from the University of Agronomic Sciences and Veterinary Medicine of Bucharest (USAMV) Research Greenhouse, Bucharest, Romania.

The plant material used was oven-dried and later subjected to grinding and extraction. Tomato flower waste (TFW) extracts were obtained by the following three environmentally friendly extraction methods: microwave-assisted extraction (MAE), ultrasound-assisted extraction (UAE), and cascade extraction (CASE), which is a sequential procedure involving UAE and MAE. All experiments were performed in triplicate. The obtained extracts were separated by filtration using Whatman No. 4 filter paper (Whatman Inc., Florham Park, NJ, USA) under vacuum. The filtrates were concentrated (using a Microvap 118 Nitrogen Evaporator, Organomation^®^, Berlin, MA, USA) and then subjected to lyophilization until further analysis.

The MAE of the TFW extract was performed using Milestone Ethos Easy equipment (Milestone Srl, Bergamo, Italy). The high power of the microwaves provided by the 2 magnetrons connected to the rotating diffuser offers a very fast heating of the samples, implicitly inducing efficient extraction of the most sensitive compounds [82]. The MAE extraction parameters were as follows: time, 32 min; solvent ethanol–water ratio, 1:1 *v*/*v*; plant-to-solvent ratio, 1:20 (*w*/*v*); temperature, 70 °C; power, 750 W; and amplitude, 50% (the sample was encoded as TFWMW). The UAE of TFW extract was performed using an ultrasonic processor (Sonics Vibra-Cell^TM^ VCX 750, SONICS & MATERIALS, INC., Newtown, CT, USA). This high-intensity system, for the extraction of bioactive compounds from plant material, involves the use of a probe that helps to produce the cavitation phenomenon. During cavitation, shock waves are created that release high energy at the level of the sample, thus resulting in an efficient extraction [83,84]. The UAE extraction parameters were as follows: time, 60 min; solvent ethanol–water, 1:1 *v*/*v*; plant-to-solvent ratio, 1:20 (*w*/*v*); temperature, 100 °C; and power, 500 W (the sample was encoded as TFWUS). The CASE extraction parameters were as follows: time, 14 min for UAE and 60 min for MAE; solvent ethanol–water ratio, 1:1 *v*/*v*; plant-to-solvent ratio, 1:20 (*w*/*v*); temperature, 70 °C for UAE and 100 °C for MAE; power, 750 W for UAE and 500W for MAE; and amplitude, 50% for UAE (the sample was encoded as TFWCAS).

The percentage of the extraction yield was expressed by the lyophilized mass extract related to the initial mass of dried plant material used for extraction.

### 4.2. Phytochemical Screening in Tomato Flower Waste Extracts

#### 4.2.1. Evaluation of Total Polyphenolic Content (TPC)

In order to determine the total polyphenolic content (TPC) of different crude extracts, the modified Folin–Ciocalteu assay [85] was used, adapted for microspectrophotometry [84]. Aliquots of hydroalcoholic extract were mixed with 1N Folin–Ciocalteu reagent, 20% Na_2_CO_3_ solution, and ultrapure water (2:1:1:4 volume ratio). The experiments were carried out in triplicate, and the absorbances were read after incubation for 30 min in the dark at room temperature, at 760 nm, using the FLUOstar^®^ Omega microplate reader (BMG LABTECH, Ortenberg, Germany). The results were expressed in milligrams of gallic acid equivalents per kilogram of dry matter (mgGAE/kg).

#### 4.2.2. Evaluation of Total Flavonoid Content (TFC)

The total flavonoid content was determined following the aluminum chloride method adapted for microspectrophotometry [86,87], from TFW, by mixing the hydroalcoholic extracts with 2% AlCl_3_ solution (1:1 volume ratio). The experiments were carried out in triplicate, and the absorbance of the samples was read after 15 min of incubation in the dark at room temperature, at 415 nm, using the FLUOstar^®^ Omega microplate reader (BMG LABTECH, Ortenberg, Germany). Using quercetin as standard, the TFC results were expressed in milligrams of quercetin equivalents per kilogram of dry matter (mgQE/kg).

### 4.3. Identification and Quantification of Polyphenolic Compounds Present in Tomato Flower Waste Extracts by UPLC

Quantitative analysis of TFW extracts was performed using an Ultra Performance Liquid Chromatography (UPLC) System (Waters Acquity UPLC^®^ I Class, Milford, MA, USA) equipped with a binary solvent manager (binary pump), sample manager, column compartment, and PDA detector. In order to perform the analytic separation process, a Zorbax Eclipse Plus C18 chromatographic column (Agilent Technologies, Santa Clara, CA, USA) was used with 4.6 × 150 mm and 5 µm of particle size. The column oven was preset at 30 °C, and the autosampler temperature was 25 °C. The mobile phase consisted of the following two solvents: solvent A containing water and solvent B containing acetonitrile. Both solvents were mixed with 0.1% formic acid. The linear gradient system started with solvent B from 10% to 100% at a flow rate of 0.8 mL/min for 30 min.

The targeted reference polyphenolic compounds were gallic acid, caffeic acid, chlorogenic acid, catechin, epicatechin, p-coumaric acid, ferulic acid, isoquercetin, rosmarinic acid, naringin, myricetin, luteolin, quercetin, and naringenin. Wavelengths of 280 nm, 320 nm, and 370 nm were used to detect the targeted polyphenolic compounds. The samples were carried out in triplicate, and the results were processed in Empower software. The 14 targeted polyphenolic compounds were identified and quantified by comparison with the retention time provided in each compound’s spectrum. The calibration curve concentrations ranged from 5 to 150 µg/mL, and the results were expressed in milligrams equivalents per kilogram dried sample (mg/kg).

### 4.4. Antioxidant Activity Evaluation of Tomato Flower Waste Extracts

#### 4.4.1. DPPH Assay

Antioxidant activity was evaluated using the DPPH (1, 1-Diphenyl-2-Picrylhydrazyl) assay, with few adaptations for the microplate method [88]. The method consisted, in brief, of mixing equal parts of the hydroalcoholic extract with 250 μM DPPH solution (1:1 volume ratio). Absorbance was read after 30 min of incubation in the dark at room temperature, at 517 nm, using the FLUOstar^®^ Omega microplate reader (BMG LABTECH, Ortenberg, Germany). The results obtained were expressed in milligrams of Ascorbic Acid Equivalents per kilogram of dry matter (mgAAE/kg). The experiments were carried out in triplicate.

#### 4.4.2. ABTS Assay

Evaluation of the antioxidant activity using the cationic radical ABTS^•+^ involved the preparation of an ABTS stock solution by mixing equal parts of 2,2′-Azino-bis (3-ethylbenzthiazoline-6-sulfonic acid) diammonium salt 7.8 mM with K_2_S_2_O_8_ 140 mM solution. After 12 h (at room temperature in the dark), the stock solution was diluted with methanol until the absorbance value assessed at 734 nm was 1.1 ± 0.02 arbitrary units. This method was an adapted version of the microplate method [89]. The experiments were carried out in triplicate and consisted of mixing and homogenizing equal parts of ABTS methanolic solution with hydroalcoholic extract (1:1 volume ratio). The reading of their absorbance was performed after incubation in the dark for 30 min at room temperature, at 734 nm, using the FLUOstar^®^ Omega microplate reader (BMG LABTECH, Ortenberg, Germany). The results were expressed in milligrams of Ascorbic Acid Equivalents per kilogram of dry matter (mgAAE/kg).

#### 4.4.3. FRAP Assay

To study the ability of the sample to reduce Fe^3+^ to Fe^2+^ ions, a FRAP solution was prepared consisting of 250 mM acetate buffer solution (pH 3.6), 10 mM 2,4,6-tris (2-pyridyl)-s-triazine (TPTZ) and 20 mM FeCl_3_, in a 10:1:1 volume ratio. A modified FRAP method was used, adapted for the microspectrophotometry technique [90]. The experiments consisted of mixing and homogenizing the hydroalcoholic extracts with the FRAP solution in a 1:4 volume ratio, which were left to incubate in the dark for 30 min at room temperature; then, their absorbance was read at 593 nm using the FLUOstar^®^ Omega microplate reader (BMG LABTECH, Ortenberg, Germany). The ferric ion reduction results were expressed in milligrams of Ascorbic Acid Equivalents per kilogram of dry matter (mgAAE/kg).

### 4.5. Phytosynthesis of Silver-Based Nanoparticles Using Tomato Flower Extracts

**Figure 14:**
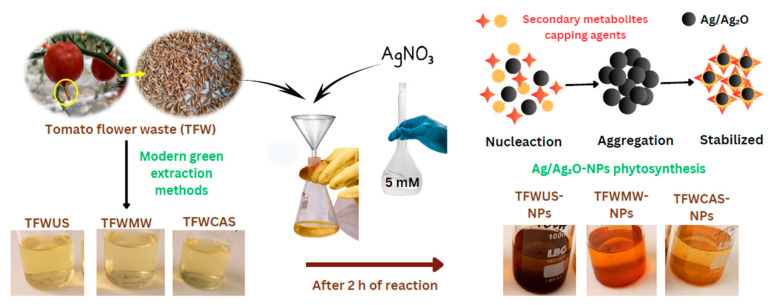
Schematic steps for the formation process of Ag/Ag_2_O-NPs.

The phytosynthesis of silver-based nanoparticles (NPs) consisted of mixing the aqueous extract of tomato flower waste (20 mg/mL) and 5 mM AgNO_3_ solution in a 1:10 volume ratio. The mixture obtained was incubated at 70 °C for 2 h. Throughout this process, the solution underwent several color changes, transitioning from colorless to yellowish and then to reddish brown before stabilizing, which confirmed the successful formation of NPs, attributed to the reduction of Ag^+^ to Ag^0^ [91,92] (Figure 14). Then, the samples were centrifuged for 30 min at 12,000 rpm.

Repeated washing with ultrapure water followed by centrifugation was performed until the supernatant color completely disappeared. The obtained NPs were lyophilized for further use. Each sample of NPs (5 mg of lyophilized powder) was resuspended in 2 mL of ultrapure water and used in the following analyses.

The yield of NPs formed, expressed as a percentage, was calculated with the following equation:

NPs yield (%) = m_NPs_/m_Ag_ × 100 (2)

where m_NPs_ represents the mass of NPs formed (g) and m_Ag_ represents the mass of Ag^+^ ions (g) used in phytosynthesis.

### 4.6. Characterization Techniques of Silver-Based Nanoparticles

#### 4.6.1. Spectrophotometric Analysis of Samples

The nanoparticles were preliminarily evaluated by UV-Vis spectrometry, using a Specord 210 Plus UV-Vis spectrophotometer (Analytik Jena, Jena, Germany, optical resolution 0.5 nm) in the 290–800 nm wavelength range.

#### 4.6.2. FTIR Spectroscopy Analysis

The Fourier transform infrared (FTIR) spectroscopy analysis was performed with a JASCO FT-IR 6300 instrument (Jasco Int. Co., Ltd., Tokyo, Japan), equipped with a Specac ATR Golden Gate (Specac Ltd., Orpington, UK) with a KRS5 lens, in the range of 400 to 4000 cm^−1^.

#### 4.6.3. XRD Analysis

The lyophilized nanoparticles were subjected to XRD analysis, in order to establish the nature of the obtained nanoparticles.

X-ray diffraction (XRD) analysis was conducted using a 9 kW Rigaku SmartLab diffractometer (Rigaku Corp., Tokyo, Japan). The instrument was operated at 45 kV and 200 mA, utilizing CuKα radiation with a wavelength of 1.54059 Å. Measurements were carried out in scanning mode 2θ/θ, covering a 2θ range from 7° to 90°. PDXL 2.7.2.0 software (Rigaku Corporation, Tokyo, Japan), for peak separation and full-width at half-maximum (FWHM) calculations, was used to analyze the diffraction data. The components were identified by comparing the obtained diffraction patterns with the International Centre for Diffraction Data (ICDD) PDF-5+ database.

#### 4.6.4. TEM Morphological Analysis

TEM studies were performed to investigate the morphology of the obtained NPs. A sample droplet was applied on a 200 mesh formvar-coated grid and allowed to air-dry. The grid sample was then analyzed using a PHILIPS EM208S operated at 80 kV accelerating voltage (Koninklijke Philips N.V., Amsterdam, The Netherlands) and visualized with a Veleta camera (EMSIS GmbH, Münster, Germany).

### 4.7. Antibacterial Potential Evaluation of Tomato Flower Extracts and Silver-Based Nanoparticles

Using the time-kill kinetics assay, the antibacterial screening of NPs and TFW extracts was carried out against pathogenic bacteria, including *Escherichia coli* (Gram-negative) and *Staphylococcus aureus* (Gram-positive). *E. coli* and *S. aureus* were grown on nutrient agar medium and then incubated for 24 h at 37 °C. A 180 μL inoculum size of approximately 1.0 McFarland diluted in liquid broth was added to each well of a 96-well microtiter plate, and then 20 μL of NPs and TFW extracts were added and incubated at 37 °C for 24 h, while measuring their OD600 every 30 min using a microplate reader (Synergy™ HTX Multi-Mode Microplate Reader, Biotek, Winooski, VT, USA). The control was bacteria without samples. The procedure was carried out in independent triplicates, and the absorbance was plotted against time.

### 4.8. Antitumoral Potential Evaluation of Tomato Flower Extracts and Silver-Based Nanoparticles

The HeLa (cervical tumor) and HT29 (colon tumor) cell lines were selected for the antitumoral potential assessment of the NPs and TFW extracts, respectively, using the MTT assay. The HeLa and HT29 cell lines were cultivated in DMEM culture medium (Sigma-Aldrich, St. Louis, MO, USA), supplemented with 10% Fetal Bovine Serum (FBS) (Sigma-Aldrich) and 1% penicillin–streptomycin (Sigma-Aldrich, Burlington, MA, USA), exposed to NPs and TFW extracts, respectively, and incubated in 5% CO_2_ at 37 °C for 24 h. H_2_O_2_ was used as a positive control, and ultrapure water was used as a negative control. Following the incubation period, the supernatant was removed, the cells were treated with MTT (Sigma-Aldrich), and the plates were incubated for 4 h at 37 °C. The purple formazan formed was dissolved in 100 μL DMSO, and the optical density was measured at 570 nm using a microplate reader (Synergy™ HTX Multi-Mode Microplate Reader, Biotek, Winooski, VT, USA).

### 4.9. Statistical Analysis

Statistical analysis of the obtained results was performed using GraphPad software package (v. 10.3.0). Statistical tests selected included analysis of variance (ANOVA) and Tukey’s test, both at a significance level of 5% (α = 0.05). Also, the correlation between TPC, TFC, and antioxidant activity was determined using the Pearson correlation coefficient.

## 5. Conclusions

The development of innovative antimicrobial and antitumor products, which provide maximum biocompatibility and prevent the emergence of resistance, is a critical aspect of modern treatments. In this context, the research undertaken in the present study on silver nanoparticles phytosynthesized by ecological methods, using extracts from tomato flower waste, has shown significant promise. These nanoparticles exhibit significant antimicrobial potential against *E. coli* and *S. aureus* pathogenic bacteria and antitumoral potential against HeLa and HT29 tumor cells, offering considerable advantages in terms of sustainability and reducing the negative impact on the environment. Therefore, these findings underline the enormous potential of ecological approaches in the development of advanced therapeutic solutions, showing potential in future medical and industrial applications.

## Figures and Tables

**Table 1:** Sample coding and extraction yield of tomato flower waste.

Sample Coding	Extraction Method	Yield (%)
TFWUS	UAE	22.57 ± 0.28 ^b^
TFWMW	MAE	26.92 ± 0.92 ^a^
TFWCAS	CASE	20.56 ± 0.49 ^c^

The results are expressed as the average of three samples (mean ± SD). TFWUS—tomato flower waste extract obtained by UAE, TFWMW—tomato flower waste extract obtained by MAE, and TFWCAS—tomato flower waste extract obtained by CASE. The superscript letter within the same column indicates significant differences detected among the studied samples using ANOVA (*p* < 0.05).

**Table 2:** Quantitative description of the polyphenolic compounds in tomato flower waste extracts by UPLC.

No.	Compound Name	RT (min)	λ_max_ (nm)	TFWUS (mg/kg)	TFWMW (mg/kg)	TFWCAS (mg/kg)
1	Gallic acid	3.07	280	260.14 ± 2.90 ^b^	324.99 ± 23.85 ^a^	198.26 ± 1.79 ^c^
2	Chlorogenic acid	5.77	320	1640.24 ± 71.77 ^a^	106.08 ± 0.57 ^b^	1728.51 ± 52.01 ^a^
3	Caffeic acid	8.33	320	36,902.34 ± 20.10 ^b^	38,720.75 ± 65.48 ^a^	32,217.04 ± 30.03 ^c^
4	p-Coumaric acid	11.08	320	408.16 ± 22.52 ^b^	469.27 ± 2.72 ^a^	401.37 ± 7.63 ^b^
5	Ferulic acid	12.47	320	338.41 ± 18.14 ^a^	367.24 ± 16.64 ^a^	329.92 ± 22.79 ^a^
6	Rosmarinic acid	15.98	320	21.32 ± 0.38 ^b^	27.01 ± 2.39 ^a^	22.44 ± 2.25 ^ab^
7	Catechin	6.29	280	291.98 ± 9.92 ^a^	287.49 ± 5.19 ^a^	253.21 ± 0.73 ^b^
8	Epicatechin	7.86	280	78.11 ± 1.75 ^b^	57.95 ± 2.72 ^c^	77.10 ± 0.46 ^a^
9	Isoquercetin	11.94	370	61.61 ± 4.04 ^a^	18.97 ± 0.90 ^b^	15.50 ± 1.48 ^b^
10	Naringin	13.70	280	45.37 ± 1.33 ^a^	28.05 ± 1.54 ^b^	26.51 ± 2.89 ^b^
11	Myricetin	16.68	370	47.37 ± 0.28 ^b^	58.94 ± 0.97 ^a^	47.21 ± 0.43 ^b^
12	Luteolin	22.20	370	245.95 ± 29.24 ^a^	105.07 ± 2.87 ^b^	107.99 ± 1.59 ^b^
13	Quercetin	22.32	370	62.13 ± 0.49 ^c^	84.69 ± 2.55 ^a^	67.48 ± 1.82 ^b^
14	Naringenin	23.83	280	33.29 ± 0.46 ^c^	69.41 ± 2.76 ^a^	47.90 ± 1.44 ^b^

RT—retention time. Superscript letters within the same row indicate significant differences detected among the studied samples using ANOVA (*p* < 0.05).

**Table 3:** Antioxidant potential of tomato flower waste extracts.

Sample	DPPH (mg AAE/kg dm)	ABTS (mg AAE/kg dm)	FRAP (mg AAE/kg dm)
TFWUS	522.89 ± 1.83 ^b^	16,707.68 ± 83.78 ^a^	5328.47 ± 67.46 ^b^
TFWMW	655.87 ± 0.05 ^a^	16,783.85 ± 11.97 ^a^	5880.65 ± 83.16 ^a^
TFWCAS	518.97 ± 1.83 ^b^	15,379.02 ± 95.75 ^b^	4594.21 ± 98.35 ^c^

The results are expressed as the average of three samples (mean ± SD). The superscript letters within the same column indicate significant differences among the studied samples (*p* < 0.05); dm = dry matter, AAE = Ascorbic Acid Equivalent.

**Table 4:** The yield of NPs formed by tomato flower waste extracts.

Sample Cod	Extract Cod	Extraction Method	Yield (%)
TFWUS-NPs	TFWUS	UAE	29.26 ^c^
TFWMW-NPs	TFWMW	MAE	34.81 ^a^
TFWCAS-NPs	TFWCAS	CASE	32.22 ^b^

UAE—ultrasound-assisted extraction, MAE—microwave-assisted extraction, CASE—cascade extraction. TFW—tomato flower waste. Extract—AgNO_3_ ratio 1:10 (*v*/*v*), extract concentration 20 mg/mL, AgNO_3_ concentration 5 mM, synthesis temperature 70 °C, time 120 min. The superscript letters within the same column indicate significant differences detected among the studied samples using ANOVA (*p* < 0.05).

**Table 5:** The crystallite size of the phytosynthesized NPs formed by tomato flower waste extracts.

Sample	Peak Position (Degrees)					FWHM (Degrees) ^1^	Crystallite Size (nm) ^1^
AgNPs	(111)	(200)	(220)	(311)	(222)		
TFWUS-AgNPs	38.12	44.19	64.62	77.36	81.44	0.57	15.44
TFWMW-AgNPs	38.08	44.34	64.41	77.46	81.39	0.59	14.86
TFWCAS-AgNPs	38.17	44.13	64.49	77.49	81.49	0.65	13.47
Ag_2_O NPs	(111)	(211)	(220)	(110)			
TFWUS-Ag_2_O-NPs	32.32	46.17	54.62	27.54		0.68	12.70
TFWMW-Ag_2_O-NPs	32.10	46.31	54.75	27.72		0.73	11.83
TFWCAS-Ag_2_O-NPs	32.19	46.09	54.78	27.68		0.85	10.18

^1^ Data for the (111) diffraction plane.

## Data Availability

All obtained datasets are included in this publication.
